# KLF15 maintains contractile phenotype of vascular smooth muscle cells and prevents thoracic aortic dissection by interacting with MRTFB

**DOI:** 10.1016/j.jbc.2024.107260

**Published:** 2024-04-04

**Authors:** Guangming Fang, Yexuan Tian, Shan Huang, Xiaoping Zhang, Yan Liu, Yulin Li, Jie Du, Shijuan Gao

**Affiliations:** Collaborative Innovation Centre for Cardiovascular Disorders, The Key Laboratory of Remodeling-Related Cardiovascular Diseases, Ministry of Education, Beijing Anzhen Hospital, Beijing Institute of Heart, Lung and Blood Vessel Diseases, Capital Medical University, Beijing, China

**Keywords:** thoracic aortic dissection, vascular smooth muscle cell, contractile phenotype, KLF15, MRTFB

## Abstract

Thoracic aortic dissection (TAD) is a highly dangerous cardiovascular disorder caused by weakening of the aortic wall, resulting in a sudden tear of the internal face. Progressive loss of the contractile apparatus in vascular smooth muscle cells (VSMCs) is a major event in TAD. Exploring the endogenous regulators essential for the contractile phenotype of VSMCs may aid the development of strategies to prevent TAD. Krüppel-like factor 15 (KLF15) overexpression was reported to inhibit TAD formation; however, the mechanisms by which KLF15 prevents TAD formation and whether KLF15 regulates the contractile phenotype of VSMCs in TAD are not well understood. Therefore, we investigated these unknown aspects of KLF15 function. We found that KLF15 expression was reduced in human TAD samples and β-aminopropionitrile monofumarate-induced TAD mouse model. Klf15KO mice are susceptible to both β-aminopropionitrile monofumarate- and angiotensin II-induced TAD. KLF15 deficiency results in reduced VSMC contractility and exacerbated vascular inflammation and extracellular matrix degradation. Mechanistically, KLF15 interacts with myocardin-related transcription factor B (MRTFB), a potent serum response factor coactivator that drives contractile gene expression. KLF15 silencing represses the MRTFB-induced activation of contractile genes in VSMCs. Thus, KLF15 cooperates with MRTFB to promote the expression of contractile genes in VSMCs, and its dysfunction may exacerbate TAD. These findings indicate that KLF15 may be a novel therapeutic target for the treatment of TAD.

Thoracic aortic dissection (TAD) is characterized by a tear in a weakened thoracic aortic wall, resulting in disruption of the medical layer, even rupture, and sudden death (1). However, the exact cause of this phenomenon is not well understood. Several risk factors for TAD development have been identified, including aortic coarctation, hypertension, and bicuspid aortic valves (2). Despite recent advances in the surgical management of TAD, few of clinically effective therapies exist to prevent or slow TAD progression, highlighting the need for novel strategies and a better understanding of the molecular events underlying the etiology of TAD and its pathogenesis (3).

Phenotypic changes of vascular smooth muscle cells (VSMCs), characterized by loss of contractile units and increased secretion of inflammatory cytokines and matrix metalloproteinase, are recognized as major initiating events of TAD development (4, 5, 6). VSMCs play an essential role in stabilizing vascular function and aortic wall integrity but switch to a synthetic, proliferative, or migratory phenotype in response to injury (7). Phenotypic switching of VSMCs contributes to the pathogenesis of several cardiovascular disorders, including pulmonary arterial hypertension, atherosclerosis, and TAD (8, 9, 10). The mechanisms underlying VSMC phenotypic switching in atherosclerosis have been well-studied (7); however, little is known about the key regulators involved in phenotypic switching of VSMCs during TAD.

To identify the potential regulators involved in VSMCs phenotypic switching and TAD progression, we use the public single-cell sequencing dataset (GSE155468) and found that Krüppel-like factor 15 (KLF15) transcripts are enriched in the VSMCs of healthy aortas but decreased in patients with aortic aneurysm. Previous research has shown that KLF15 overexpression inhibits TAD formation (11). Mice with KLF15 deficiency develop aortic aneurysms in a p53-dependent fashion (12). These studies support the protective role of KLF15 in TAD. Furthermore, several lines of evidence implicate the role of KLF15 in maintaining VSMCs function during the pathological conditions, including atherosclerosis and postinjury neointima formation (13, 14). However, the mechanism of action of KLF15 on the contractile phenotype of VSMCs during TAD progression requires further study.

Therefore, in this study, we investigate whether KLF15 maintains the contractile phenotype of VSMCs in TAD and explore the underlying mechanism through experiments on human TAD samples and a β-aminopropionitrile monofumarate (BAPN)-induced TAD mouse model.

## Results

### KLF15 is downregulated in VSMCs from human and mice TAD tissues

**Figure 1:**
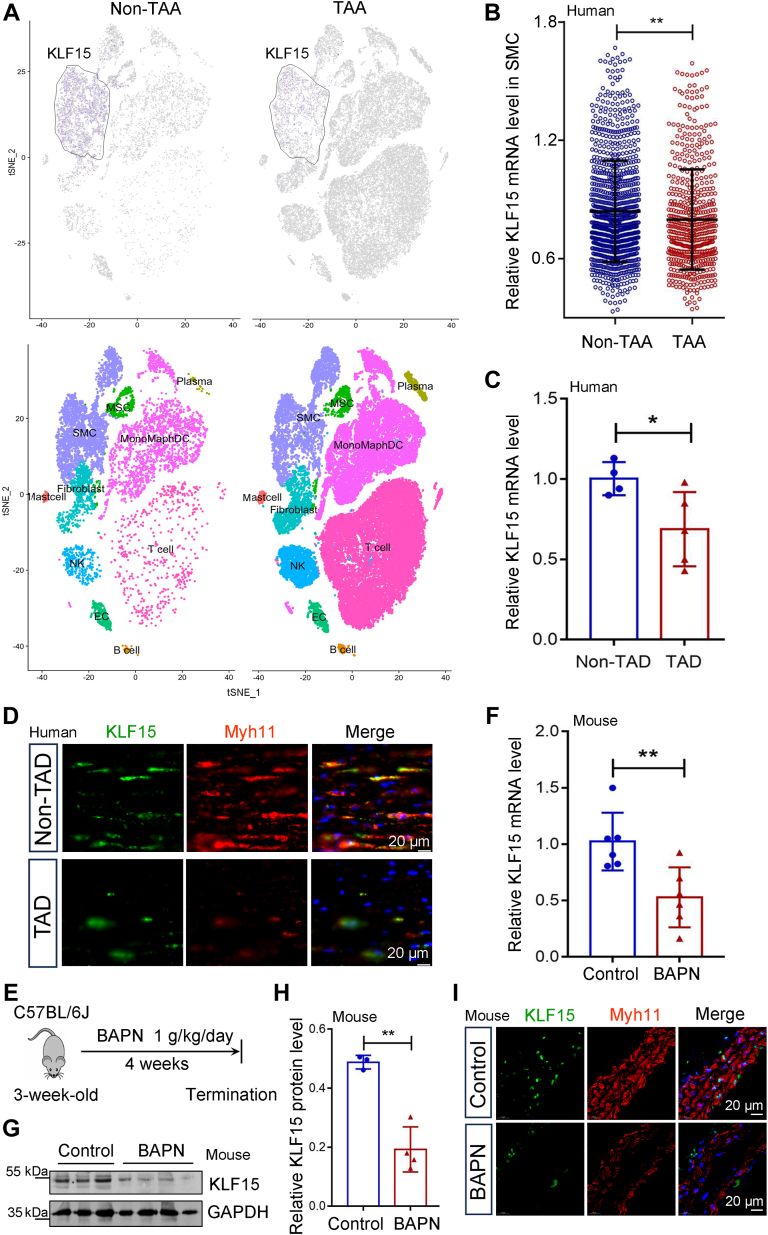
**KLF15 is downregulated in human and mice TAD tissues.***A* and *B*, a public single-cell sequencing dataset (GSE155468) of thoracic aortic aneurysm samples (TAA) and control aorta samples (non-TAA) was downloaded from the Gene Expression Omnibus database. *A*, t-SNE plot showing cell-type clusters. *B*, relative KLF15 expression levels in SMCs (n = 1031 from three non-TAA samples, n = 581 from eight TAA samples). ∗∗*p* < 0.01, by Mann-Whitney *U* test. *C*, relative mRNA levels of KLF15 in aorta tissues from human non-TAD controls (n = 4) and TAD patients (n = 5). Data are representative of three independent experiments. ∗*p* < 0.05, by Student *t* test. *D*, representative images of KLF15 expression by immunofluorescence staining of aorta tissues from human non-TAD controls (n = 4) and TAD patients (n = 5). *Red*, KLF15; *Green*, Myh11, the VSMC contractile marker; *Blue*, DAPI. *E*–*I*, aortic tissues from non-TAD control and BAPN-induced TAD model mice. *E*, schematic illustration of BAPN-induced TAD model. Three-week-old male C57BL/6J mice were administered BAPN (1 g/kg/day) in their drinking water for 4 weeks to induce the TAD model. *F*, relative mRNA levels of KLF15 (n = 6 per group). Data are representative of three independent experiments. ∗∗*p* < 0.01, by Student *t* test. *G*, Western blotting analysis of KLF15 protein levels. Data are representative of three independent experiments. *H*, protein levels of KLF15 were quantified using Image J and normalized to that of GAPDH (n = 3 for control, n = 4 for BAPN group). ∗∗*p* < 0.01, by Student *t* test. *I*, representative immunofluorescence images of KLF15 (*green*) and Myh11 (*red*). DAPI was used to stain the nucleus (*blue*). Data are representative of three independent experiments. BAPN, β-aminopropionitrile monofumarate; DAPI, 4′,6-diamidino-2-phenylindole; KLF, Krüppel-like factor; MYH11, myosin-11; TAD, thoracic aortic dissection; VSMCs, vascular smooth muscle cells.

After examining the public single-cell sequencing dataset (GSE155468) (15), we found that KLF15 transcripts were enriched in VSMCs (Fig. 1*A*) and decreased in the VSMCs of patients with thoracic aortic aneurysm(TAA) compared to healthy controls (Fig. 1*B*). Reverse transcription quantitative real-time PCR (RT-qPCR) analysis also revealed downregulation of KLF15 in the aortas of patients with TAD (Fig. 1*C*). Immunofluorescence costaining of KLF15 and the VSMC marker myosin-11 (Myh11) showed that KLF15 expression was decreased in the VSMCs of patients with TAD compared to those of non-TAD controls (Fig. 1*D*). We then generated a classical BAPN-induced TAD model in mice (9, 16, 17) to explore the changes in KLF15 levels (Fig. 1*E*). Similarly, both KLF15 mRNA and protein levels were reduced in BAPN-treated TAD tissues (Fig. 1, *F*–*H*). Moreover, KLF15 was costained with Myh11 in the VSMCs of non-TAD control aortic specimens, whereas their intensities were lower in the VSMCs of BAPN-induced TAD tissues (Fig. 1*I*).

### KLF15 deficiency aggravates TAD formation *in vivo*

**Figure 2:**
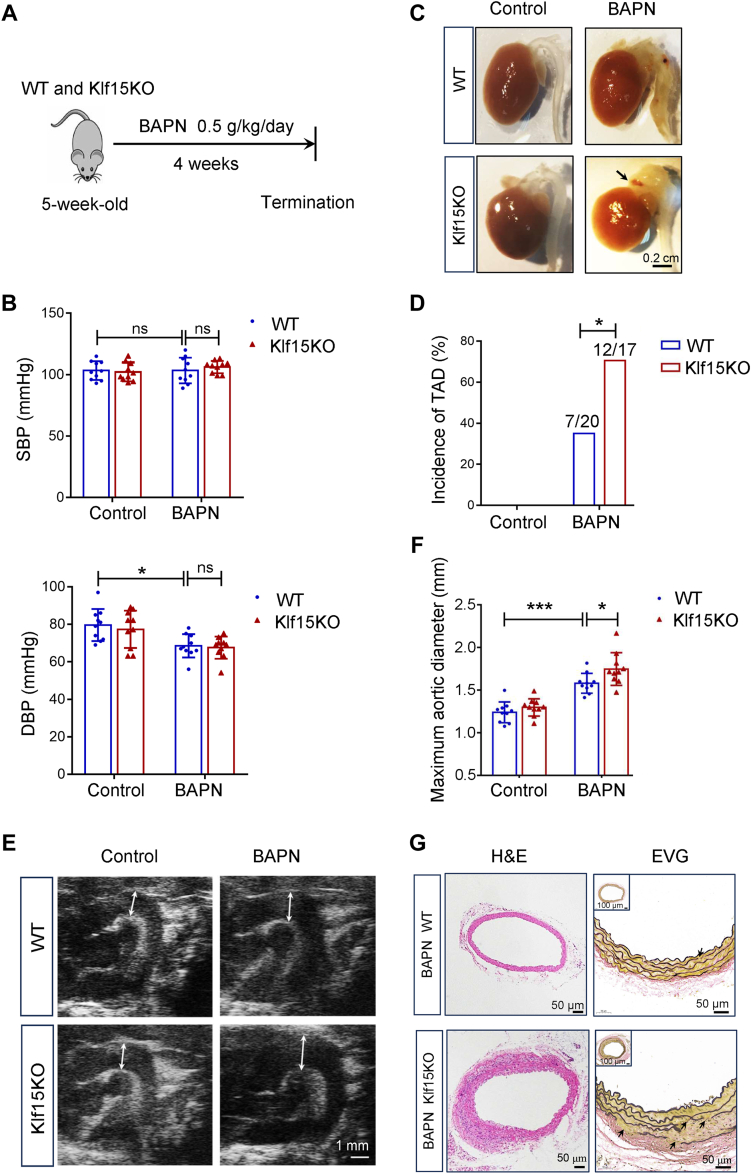
**KLF15 deficiency aggravates BAPN-induced TAD in mice.***A*, five-week-old male WT and Klf15KO mice were administered BAPN (0.5 g/kg/day) for 4 weeks to evaluate whether Klf15KO aggravates BAPN-induced TAD formation. *B*, blood pressure in WT and Klf15KO mice with or without BAPN administration (n = 10 per group). ∗*p* < 0.05, by Two-Way ANOVA with Tukey post-hoc test. *C*, representative photographs of aortas from WT and Klf15KO mice with or without BAPN administration (n = 17–20 per group). *D*, incidence of TAD (n = 17–20 per group). ∗*p* < 0.05, by Fisher’s exact test. *E*, representative ultrasound images of ascending aortas and artic arches (n = 10 per group). *F*, maximal aortic internal diameters as determined by Vevo 2100 software based on the ultrasound images (n = 10 per group). ∗*p* < 0.05, ∗∗∗*p* < 0.001, by two-way ANOVA with Tukey post-hoc test. *G*, representative images of thoracic aorta sections stained with H&E and elastic Van Gieson (EVG) (n = 10 per group). *Black arrow*: fragmented elastin. BAPN, β-aminopropionitrile monofumarate; DBP, diastolic blood pressure; KLF, Krüppel-like factor; ns, no significant difference; SBP, systolic blood pressure; TAD, thoracic aortic dissection.

Here, we investigated whether KLF15 deficiency aggregates TAD injury. Five-week-old male wildtype (WT) and Klf15 knockout (KO) mice were given BAPN (0.5 g/kg/day) for 28 days (Fig. 2*A*) to induce TAD model. BAPN administration has no effect on systolic blood pressure (SBP) but slightly reduced diastolic blood pressure (DBP) in both WT and Klf15KO mice because of increased aortic stiffness (Fig. 2*B*). During BAPN treatment, the incidence of TAD in Klf15KO mice (70%) was higher than that in WT mice (35%) (Fig. 2, *C* and *D*). KLF15 deficiency aggravated aortic dilation in BAPN-induced WT mice compared to that in the control group, as evidenced by the maximal aortic diameter measured from the vascular ultrasound images (Fig. 2, *E* and *F*). Histological analysis of the aortic wall with hematoxylin and eosin (H&E) and elastic Van Gieson (EVG) staining showed dissection (intense cell infiltration and erythrocyte extravasation) and elastin fragmentation in Klf15KO mice (Fig. 2*G*).

**Figure 3:**
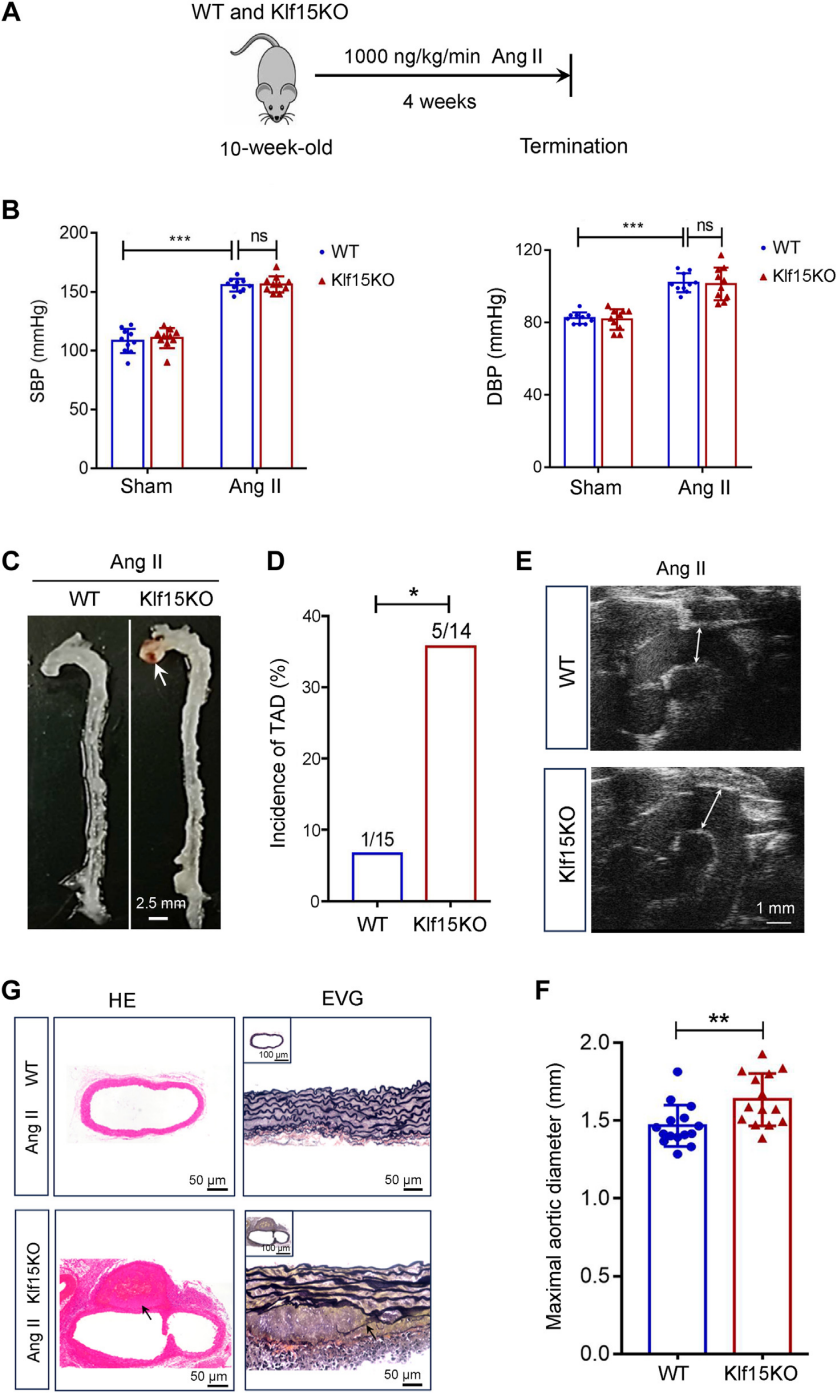
**KLF15 deficiency promotes Ang II-induced TAD formation in mice.***A*, ten-week-old male WT and Klf15KO mice were infused with angiotensin II (Ang II) (1000 ng/kg/min) for 4 weeks to assess the effect of Klf15KO on Ang II-induced TAD. *B*, blood pressure in WT and Klf15KO mice infused with saline or Ang II (n = 10 per group). ∗∗∗*p* < 0.001, by two-way ANOVA with Tukey post-hoc test. *C*, representative photograph of aortas from WT and Klf15KO mice after Ang II infusion (n = 14–15 per group). *D*, incidence of TAD (n = 15 in WT group, n = 14 in Klf15KO group). ∗*p* < 0.05, by Fisher’s exact test. *E*, representative ultrasound images of ascending aortas and artic arches (n = 10 per group). *F*, maximal aortic diameter as determined by Vevo 2100 software based on the ultrasound images (n = 15 in WT group, n = 14 in Klf15KO group). ∗∗*p* < 0.01, by Student *t* test. *G*, representative H&E-stained aortas illustrating erythrocyte extravasation (*black arrow*), and EVG-stained aortas illustrating elastin fragmentation (*black arrow*) (n = 6 per group). BAPN, β-aminopropionitrile monofumarate; DBP, diastolic blood pressure; KLF, Krüppel-like factor; ns, no significant difference; SBP, systolic blood pressure; TAD, thoracic aortic dissection.

To confirm the effects of KLF15 deficiency on TAD formation, we performed further experiments using an angiotensin II (Ang II) infusion model (Fig. 3*A*). Ang II increased SBP and DBP similarly in WT and Klf15KO mice (Fig. 3*B*). Ang II-only infusion in mice is commonly used to induce hypertension and barely induces TAD formation (18, 19). Klf15KO mice that underwent Ang II-only infusion showed a higher incidence of TAD (35.7%, 5/14) and an increase in the maximal aortic diameter compared to WT mice (6.7%, 1/15) (Fig. 3, *C*–*F*). Consistent with the increased incidence of TAD in Klf15KO mice, H&E and EVG staining revealed erythrocyte extravasation and elastin degradation, which resulted in disconnection of the medial walls (Fig. 3*G*). Taken together, these data indicate that KLF15 deficiency aggravates TAD *in vivo*.

### KLF15 deficiency promotes VSMC phenotypic switching *in vivo* and *in vitro*

**Figure 4:**
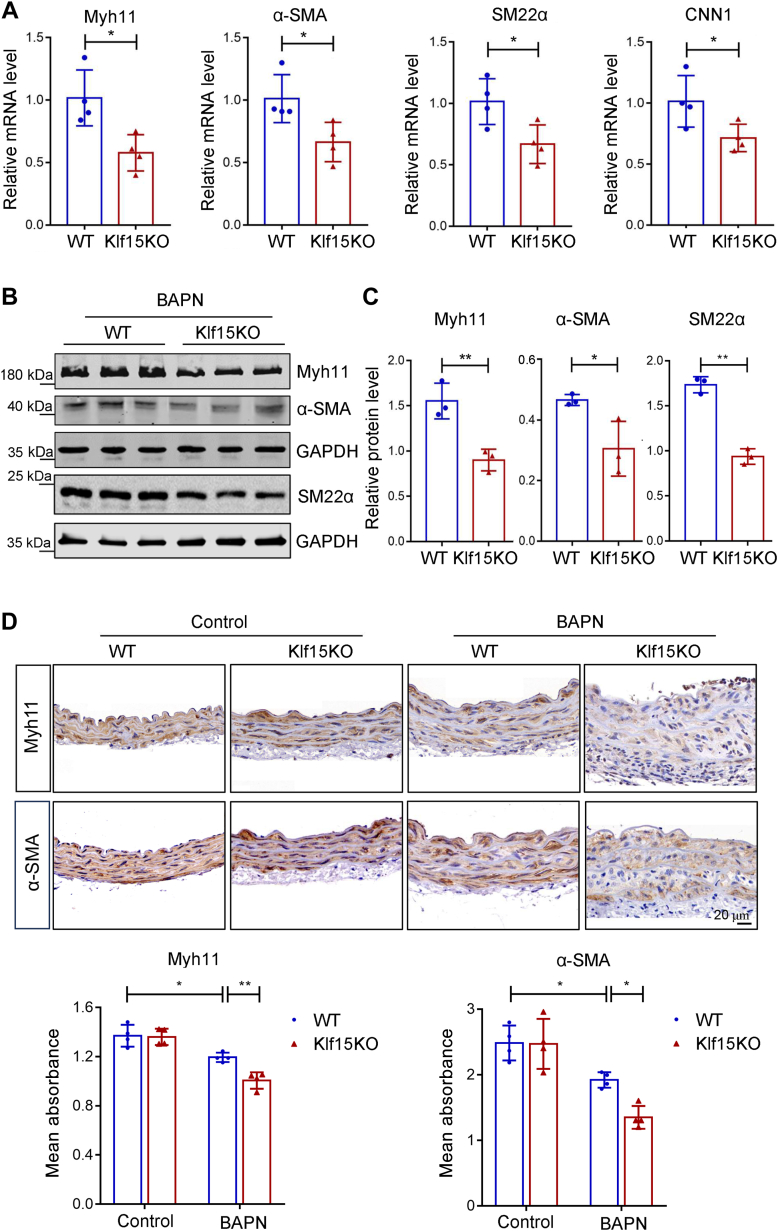
**Reduced VSMC contractile markers in Klf15KO aortas.** Five-week-old male WT and Klf15KO mice were administered BAPN for 28 days. *A*, relative mRNA levels of VSMC contractile markers Myh11, α-SMA, SM22α, and CNN1 (n = 4 per group). ∗*p* < 0.05, by Student *t* test (Myh11, SM22α, and CNN1) or by Mann-Whitney *U* test (α-SMA). Data are representative of three independent experiments. *B*, Western blotting analysis of protein levels of VSMC contractile proteins Myh11, α-SMA, and SM22α in WT and Klf15KO thoracic aortas. Data are representative of three independent experiments. *C*, relative protein levels of Myh11, α-SMA, and SM22α normalized to that of GAPDH (n = 3). ∗*p* < 0.05, ∗∗*p* < 0.01, by Student *t* test. *D*, representative immunohistochemical staining and quantification of Myh11 and α-SMA in thoracic aorta samples from WT and Klf15KO mice (n = 4 per group). ∗*p* < 0.05, ∗∗*p* < 0.01, by two-way ANOVA with Tukey post-hoc test. α-SMA, α-smooth muscle actin; BAPN, β-aminopropionitrile monofumarate; CNN1, calponin1; KLF, Krüppel-like factor; MYH11, myosin-11; SM22α, smooth muscle 22 alpha; VSMCs, vascular smooth muscle cells.

Given that loss of the contractile apparatus in VSMCs is a major event leading to TAD (5, 6), we first measured the levels of contractile markers in BAPN-treated WT and Klf15KO aortas. When compared with those from WT mice, RT-qPCR analysis of ascending aorta samples from Klf15KO mice showed reduced expression of contractile genes, including Myh11, α-smooth muscle actin (α-SMA), smooth muscle 22 alpha (SM22α), and calponin1 (Fig. 4*A*). We also detected a slight downregulation of Myh11, α-SMA, and SM22α proteins in Klf15KO mice by Western blotting analysis and immunohistochemical staining (Fig. 4, *B*–*D*).

**Figure 5:**
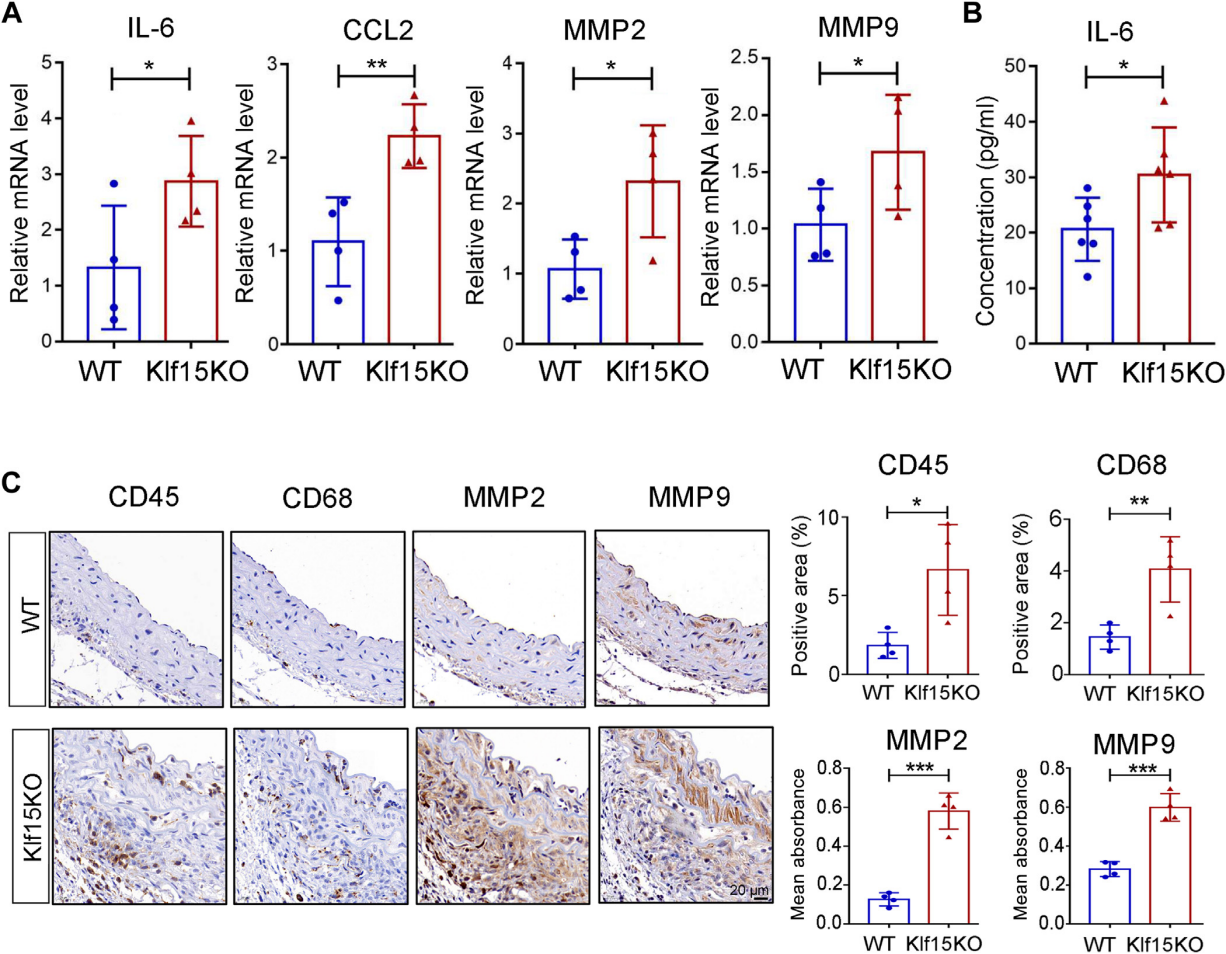
**KLF15 deletion results in upregulation of inflammation cytokines and MMPs in the aorta.** Five-week-old male WT and Klf15KO mice were administered BAPN for 28 days. *A*, relative mRNA levels of inflammatory cytokines IL-6, CCL2, and matrix metalloproteinases MMP2 and MMP9 (n = 4 per group). ∗*p* < 0.05,∗∗*p* < 0.01, by Student *t* test. Data are representative of three independent experiments. *B*, ELISA analysis of plasma IL-6 levels from BAPN-treated WT and Klf15KO mice (n = 6 per group). ∗*p* < 0.05, by Student *t* test. *C*, representative immunohistochemical staining and quantification of leukocyte marker CD45, macrophage marker CD68, and matrix metalloproteinases MMP2 and MMP9 in the thoracic aorta samples from BAPN-treated WT and Klf15KO mice (n = 4 per group). ∗*p* < 0.05, ∗∗*p* < 0.01, ∗∗∗*p* < 0.001, by Student *t* test. BAPN, β-aminopropionitrile monofumarate; KLF, Krüppel-like factor.

VSMCs phenotypic switching from a contractile to synthetic inflammatory phenotype may induce the expression of proinflammation cytokines and MMPs, which contribute to the pathogenesis of TAD (8). Thus, we compared the levels of proinflammatory cytokines and MMPs in WT and Klf15KO aortas after BAPN administration. RT-qPCR revealed higher expression of the proinflammatory cytokines, IL-6 and CCL2, and matrix metalloproteinases, MMP2 and MMP9, in BAPN-treated Klf15KO aortas than in WT mice (Fig. 5*A*). Increased IL-6 secretion in Klf15KO aortic rings upon BAPN administration was also observed using ELISA (Fig. 5*B*). Consistently, CD45^+^ leukocyte and CD68^+^ macrophage infiltration was enhanced in the aortas of Klf15KO mice. Upregulation of MMP2 and MMP9 in the aortas of Klf15KO mice was confirmed by immunohistochemical staining (Fig. 5*C*).

**Figure 6:**
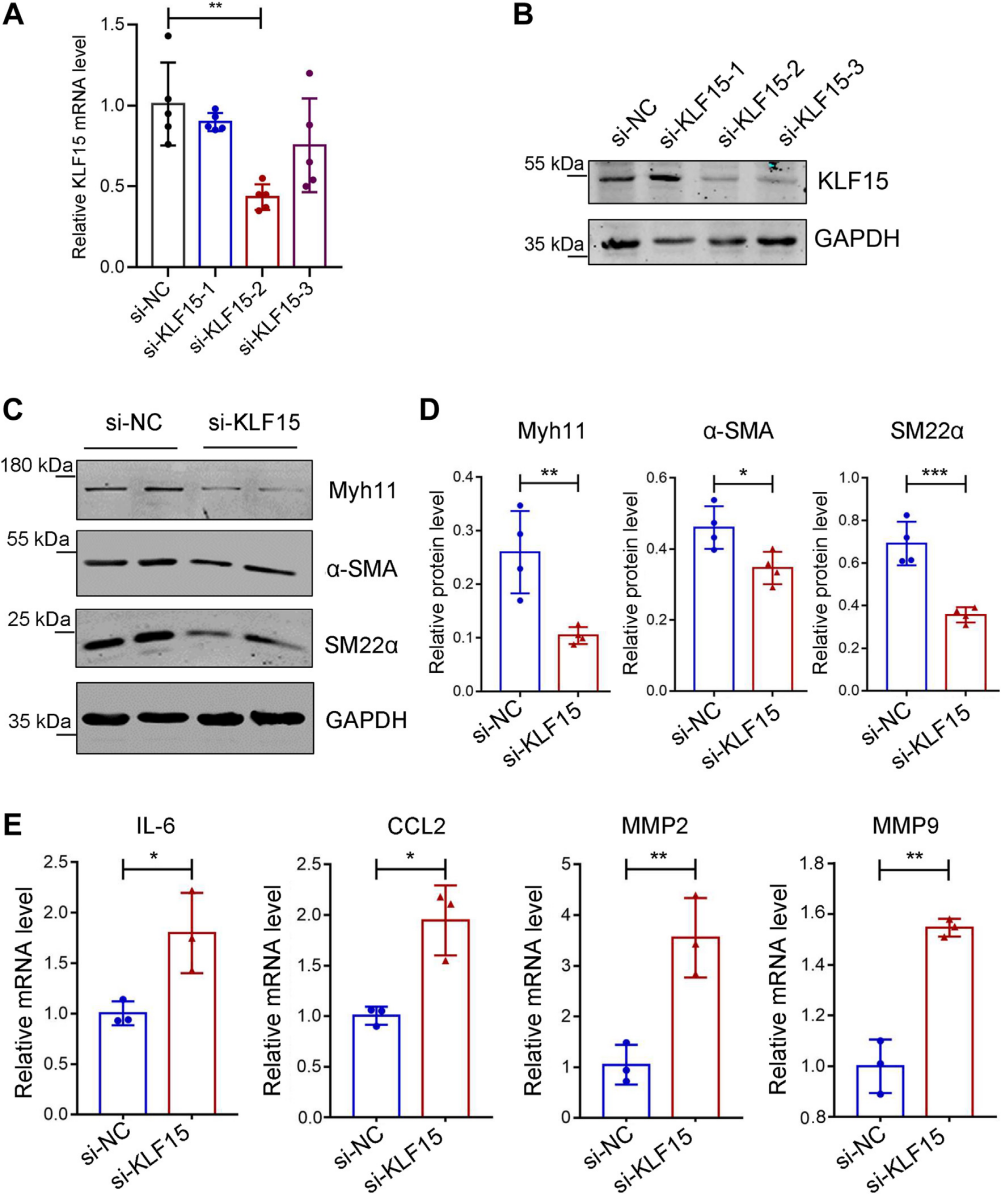
**KLF15 knockdown induces VSMC phenotypic switching *in vitro*.***A* and *B*, HEK293T cells were transfected with KLF15-targeting (si-KLF15) or control siRNA (si-NC). *A*, RT-qPCR was used to evaluate KLF15 knockdown efficiency (n = 5 per group). ∗∗*p* < 0.01, by one-way ANOVA with Sidak’s multiple comparison test. *B*, Western blotting was used to evaluate KLF15 knockdown efficiency. Data are representative of three independent experiments. *C*, HASMCs were transfected with si-NC or si-KLF15-2 for 72 h. Western blotting analysis of contractile markers Myh11, α-SMA, and SM22α. Data are representative of two independent experiments. *D*, relative protein levels of Myh11, α-SMA, and SM22α normalized to that of GAPDH (n = 4 from two independent experiments). ∗*p* < 0.05,∗∗*p* < 0.01, ∗∗∗*p* < 0.001 by Mann-Whitney *U* test (Myh11) or by Student *t* test (α-SMA and SM22α). *E*, HASMCs were transfected with si-NC or si-KLF15-2 for 36 h followed by serum starvation for 12 h, then stimulated with AngII (1 μM) for 24 h. RT-qPCR analysis of relative mRNA levels of inflammatory cytokines IL-6, and CCL2 and matrix metalloproteinases MMP2 and MMP9 (n = 3 per group). ∗*p* < 0.05, ∗∗*p* < 0.01, by Student *t* test. Data are representative of three independent experiments. α-SMA, α-smooth muscle actin; Ang II, angiotensin II; HASMCs, human aortic smooth muscle cells; KLF, Krüppel-like factor; MYH11, myosin-11; SM22α, smooth muscle 22 alpha; VSMCs, vascular smooth muscle cells.

We next investigated the effects of KLF15 on the phenotypic switching of VSMCs *in vitro*. KLF15 siRNA was used to knockdown KLF15 (Fig. 6, *A* and *B*). KLF15 knockdown in human aortic smooth muscle cells (HASMCs) caused a mild downregulation of the contractile proteins Myh11 and SM22α and a negligible change in the α-SMA protein level (Fig. 6, *C* and *D*). Moreover, upon Ang II stimulation, KLF15 knockdown upregulated the mRNA levels of the proinflammatory cytokines IL-6 and CCL2 and the matrix metalloproteinases MMP2 and MMP9 in HASMCs (Fig. 6*E*). These *in vivo* and *in vitro* data suggested that KLF15 deficiency is involved in the phenotypic switching of VSMCs from a contractile to synthetic inflammatory phenotype.

### Myocardin-related transcription factor B is a novel binding protein of KLF15

**Figure 7:**
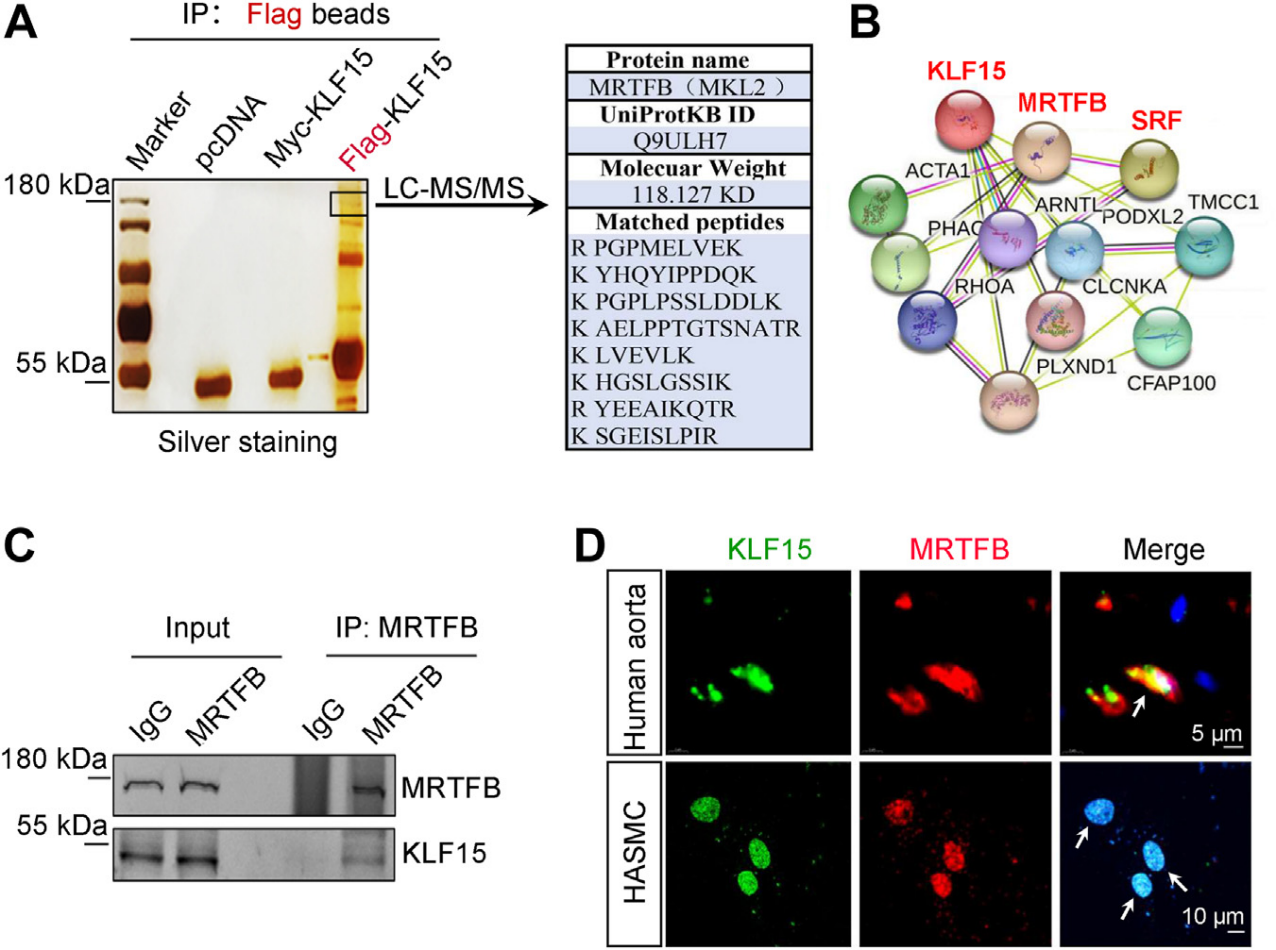
**KLF15 interacts with MRTFB.***A*, HEK293T cells were transfected with the pcDNA control, Myc-KLF15 control, or Flag-KLF15 plasmid. Cell lysates were collected for co-immunoprecipitation, silver staining, and mass spectrometry analyses. *B*, protein–protein interaction network for KLF15 and MRTFB derived from the STRING database. *C*, co-immunoprecipitation assay of the interaction between endogenous KLF15 and MRTFB in HASMCs. The HASMCs lysate was immunoprecipitated with an anti-MRTFB antibody or the control IgG. Precipitates were analyzed by immunoblotting with anti-KLF15 and anti-MRTFB antibodies. Data are representative of three independent experiments. *D*, immunofluorescence analysis of the colocalization of KLF15 (*green*) and MRTFB (*red*) in human aorta samples and HASMCs. Data are representative of three independent experiments. HASMCs, human aortic smooth muscle cells; KLF, Krüppel-like factor; MRTFB, myocardin-related transcription factor B.

To understand the mechanism by which KLF15 regulates the contractile phenotype of VSMCs, we performed an immunoprecipitation assay to identify the binding proteins of KLF15. Lysates collected from Flag-KLF15-transfected cells were immunoprecipitated using anti-FLAG beads, followed by LC-MS/MS analysis (Fig. 7*A*). Among the identified proteins, myocardin-related transcription factor B (MRTFB) was noticed because of its function as a potent transcriptional coactivator of serum response factor (SRF) that drives contractile gene expression (20). The potential interactions between KLF15 and MRTFB were evaluated using the STRING database (Fig. 7*B*). We verified the interaction between endogenous KLF15 and MRTFB in HASMCs using a co-immunoprecipitation assay (Fig. 7*C*). Finally, nuclear colocalization of KLF15 and MRTFB was observed both *in vitro* in HASMCs and *in vivo* in human aortas using immunofluorescence confocal microscopy (Fig. 7*D*).

### KLF15 cooperates with MRTFB to promote the expression of contractile genes

**Figure 8:**
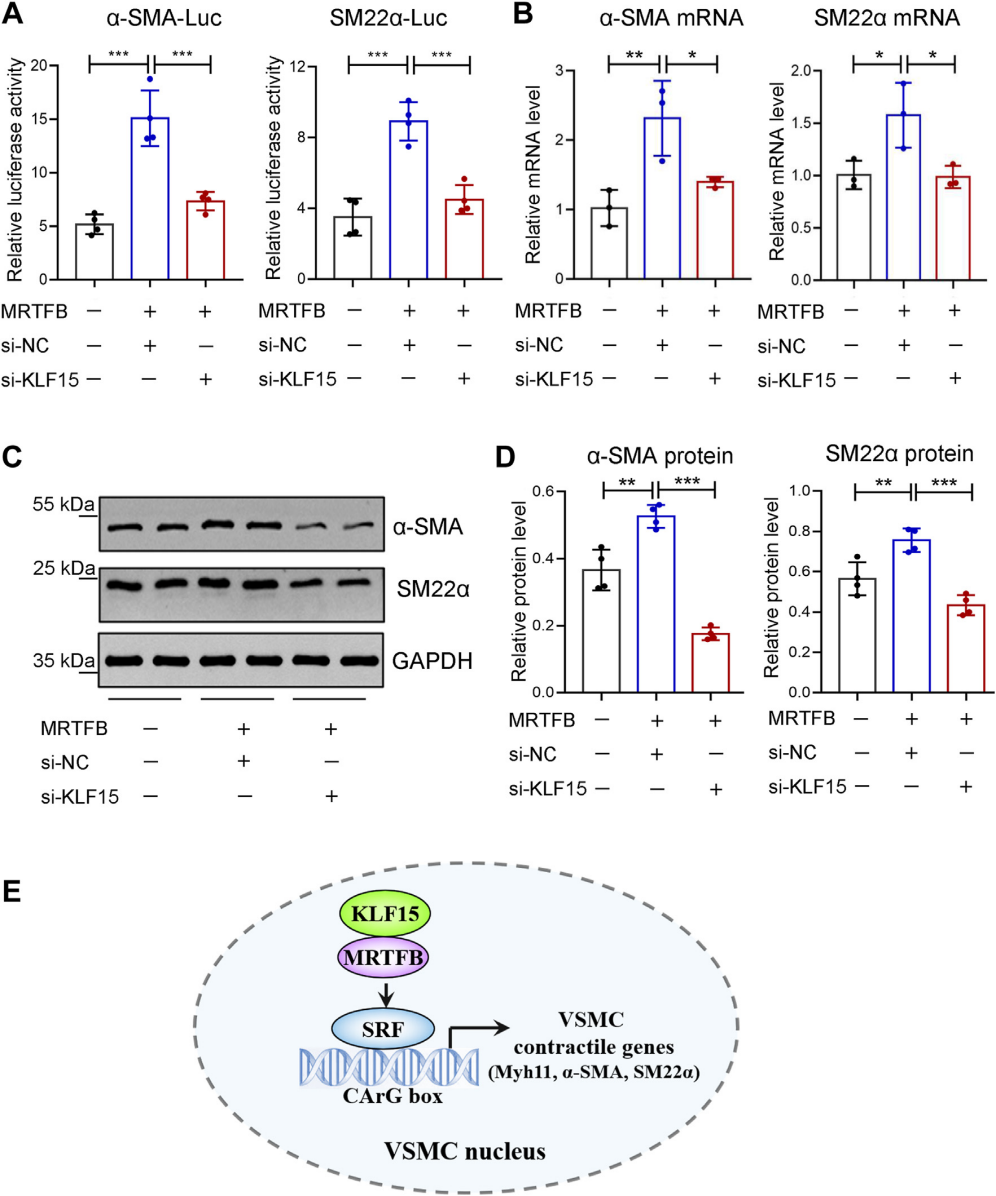
**KLF15 knockdown suppresses MRTFB-induced VSMC contractile markers expression.***A*, HASMCs were cotransfected with KLF15 siRNAs, MRTFB plasmid, and either the α-SMA-Luc or SM22α-Luc reporter plasmid. Relative luciferase activity of α-SMA-Luc or SM22α-Luc was normalized to the internal control *Renilla* luciferase (n = 4 per group). ∗∗∗*p* < 0.001, by one-way ANOVA with Sidak’s multiple comparison test. Data are representative of three independent experiments. *B*–*D*, HASMCs were cotransfected with si-NC or si-KLF15-2 and the MRTFB plasmid. *B*, relative mRNA levels of α-SMA and SM22α (n = 3 per group). ∗*p* < 0.05, ∗∗*p* < 0.01, by one-way ANOVA with Tukey’s multiple comparison test. Data are representative of three independent experiments. *C*, Western blotting analysis of α-SMA and SM22α protein levels. Data are representative of two independent experiments*. D*, relative protein levels of α-SMA and SM22α, normalized to that of GAPDH (n = 4 from two independent experiments). ∗*p* < 0.05, ∗∗*p* < 0.01, ∗∗∗*p* < 0.001, by one-way ANOVA with Tukey’s multiple comparison test. *E*, schematic illustration of the role of KLF15–MRTFB interaction in maintaining the VSMC contractile phenotype. α-SMA, α-smooth muscle actin; HASMCs, human aortic smooth muscle cells; KLF, Krüppel-like factor; MRTFB, myocardin-related transcription factor B; SM22α, smooth muscle 22 alpha; VSMCs, vascular smooth muscle cells.

We next investigated whether the interaction between KLF15 and MRTFB was involved in the contractile phenotype of VSMCs. Overexpression of MRTFB upregulated α-SMA-luc and SM22α-luc activities. KLF15 silencing repressed the MRTFB-induced transactivation of α-SMA-luc and SM22α-luc (Fig. 8*A*). Accordingly, MRTFB-induced α-SMA and SM22α expression was repressed by KLF15 silencing at both the mRNA and protein levels (Fig. 8, *B*–*D*). These data revealed the regulatory role of KLF15–MRTFB interaction in maintaining the contractile phenotype of VSMCs (Fig. 8*E*).

## Discussion

These findings confirm that KLF15 deficiency increases the risk of TAD, as supported by data from two TAD models in Klf15KO mice. Our data further revealed that KLF15 deficiency promoted VSMC phenotypic switching characterized by the loss of contractile units and increased expression of proinflammatory cytokines and MMPs, which may exacerbate TAD development. We also found that KLF15 maintained the contractile phenotype of VSMCs by binding to MRTFB. This study highlights the critical role of KLF15 in VSMCs homeostasis and suggests that KLF15 dysfunction may exacerbate TAD.

KLF15 overexpression protects against BAPN-induced AD rupture, and KLF15 inhibits CTGF expression in primary fibroblasts (11). These results suggest a role for KLF15 in fibroblasts. In this study, we focused on the functions of KLF15 in VSMCs as it is highly expressed in the VSMCs of healthy aortas but downregulated in the VSMCs of patients with aortic aneurysms, as evidenced by the public single-cell sequencing dataset (GSE155468). Previous studies have also suggested a causal role for KLF15 deficiency in aortopathy, where its deficiency leads to hyperacetylation of p53 when subjected to pressure overload (12); however, its roles in VSMCs phenotypic switching is unknown. In this study, we observed VSMCs phenotypic switching from a contractile to a dedifferentiated synthetic state in BAPN-treated Klf15KO mice. The functions of KLF15 in VSMCs have also been indentified in pathological states, such as atherosclerosis and postinjury neointima formation (13, 14). Smooth muscle–specific deficiency of KLF15 augmentes inflammatory signaling and accelerates atherogenesis in atherosclerosis. The underlying mechanism involves interactions between KLF15 and histone acetyltransferase p300, which attenuates p300-dependent NF-κB acetylation and inhibits NF-κB activation (14). In addition, the group reported that KLF15 inhibits VSMC proliferation and migration as well as postinjury neointima formation in a postangioplasty restenosis model, although the mechanism remains unclear (13). Collectively, the effects of KLF15 on VSMC biology depend on its interaction with specific factors *in vivo* in various physiological and pathological states.

VSMCs show distinct phenotypes in cardiovascular diseases. In TAD lesions, VSMCs exhibit a loss of contractile proteins, degradation of the extracellular matrix, and accumulation of inflammation, resulting in a weakened aortic wall (9), which is consistent with the results observed in this study. A recent study reported that downregulation of Talin-1 in TAD induces VSMCs proliferation and migration, which may be involved in the pathological process of TAD (21). However, the roles of VSMCs proliferation and migration in TAD are retained to be clarified. In atherosclerotic lesions, VSMCs migrate to the endothelium where they proliferate and secrete collagen, adhesion molecules, and inflammatory cytokines, leading to the formation of fibrous caps or inflammatory-type VSMCs (7). However, increased VSMC contraction can also contribute to cardiovascular diseases. For example, activation of the IP3 signaling pathway in VSMCs contributes to increased vasoconstriction and cardiac afterload during heart failure progression (22). Therefore, it is important to elucidate the exact mechanisms underlying VSMC dysfunction in response to diverse environmental stimuli. Transcription factors and epigenetics are the main regulatory mechanisms responsible for phenotypic switching in VSMCs (23). In this study, using immunoprecipitation and LC-MS/MS analysis, we identified that KLF15 interacts with MRTFB, a potent transcriptional coactivator of SRF. KLF15 silencing prevented the MRTFB-induced transactivation of contractile genes *in vitro*, and KLF15 deficiency reduced the expression of contractile genes in the TAD model *in vivo*.

MRTFB, MRTFA, and myocardin are myocardin-related transcription factors (MRTFs), which function as essential transcriptional coactivators promoting interactions between SRF homodimers and CArG elements within the promoters of contractile genes (24). As MRTFs do not possess DNA-binding activity, they activate VSMC-specific genes through physical interactions with SRF (25). Factors that influence the nuclear translocation of MRTFs and formation of the myocardin–SRF–CArG complex can regulate VSMC contractile genes expression. For example, bone morphogenetic protein induces the nuclear translocation of MRTFA or MRTFB and their recruitment to the VSMC-specific CArG element to activate transcription (26). In contrast, KLF4 represses the expression of VSMC contractile genes by suppressing myocardin–SRF–CArG interactions (27). Although MRTFs play essential roles in the differentiation of VSMCs, they may also act as mediators of pathological intima formation and the hypertrophic response of the heart. Elevated cAMP levels repress MKL1 (MRTFA) and MKL2 (MRTFB) nuclear translocation and inhibits the proliferation and migration of VSMCs. MRTFA deletion in mice reduces pathological intima formation (28). MRTFA and MRTFB also enhance the SRF-dependent transcription of the hypertrophic gene atrial natriuretic factor in a CArG box-dependent manner in cardiomyocytes (29). KLF15 represses the transcriptional activity of MRTFs in cardiomyocytes and prevents Ang II-indcued cardiac hypertrophy by interacting with MRTF (30). Conversely, our data indicate that KLF15 increases MRTFB-induced transcription in VSMCs. Therefore, it is important to understand the molecules and mechanisms that mediate the activation or inhibition of MRTFB by KLF15 in different cells.

The present study has several limitations. First, there is a need to clarify the contribution of KLF15 in TAD using VSMCs-specific KLF15 knockout mice. Second, we were unable to elucidate the intricate mechanisms underlying the dynamic association and dissociation between KLF15 and MRTFB to maintain contractile gene expression when their levels remain unchanged. Proximity ligation assay could offer valuable insights into visualizing the interactions between KLF15 and MRTFB within cellular environments. Third, the treatment values that target KLF15 to slow down TAD progression have not yet been evaluated.

In conclusion, our study identified a fundamental role for KLF15 in maintaining the contractile phenotype of VSMCs and preventing vascular injury during TAD development. In addition, this research extends previous observations on the functions of KLF15 in VSMC homeostasis and provides a reference for identifying novel therapeutic targets for TAD.

## Experimental procedures

### Reagents

BAPN, Ang II, and FLAG Immunoprecipitation Kits were purchased from Sigma. The Mouse IL-6 ELISA Kit was purchased from R&D Systems. The dual-Luciferase Reporter Assay System was from Promega. Human KLF15-targeting siRNAs were purchased from RIBOBIO. The plasmid transfection reagent Lipofectamine 3000 and siRNA transfection reagent Lipofectamine RNAiMAX were ordered from Thermo Fisher. The antibodies are listed in Table S2.

### Animals and TAD models

Animal studies were approved by the Animal Subjects Committee of Capital Medical University. WT and Klf15KO C57BL/6J mice (31) were bred and housed under pathogen-free conditions at the animal facilities of Beijing Anzhen Hospital, Capital Medical University. To establish the TAD model, 3-week-old male C57BL/6J mice were administered BAPN (1 g/kg/day) in water for 28 days (16). Owing to the high incidence of aortic dissection (up to 100%), rupture, and the high death rate as reported in this model (17, 32), to evaluate whether Klf15KO aggravates BAPN-induced TAD formation, 5-week-old male WT and Klf15KO mice were administered BAPN (0.5 g/kg/day) for 28 days (17). To assess the effect of KLF15 knockout on Ang II-induced TAD, 10-week-old male WT and Klf15KO mice were infused with Ang II (1000 ng/kg/min) for 28 days (19).

### Human TAD tissues

Dissected and nondissected ascending aorta samples were collected from five TAD patients at Beijing Anzhen Hospital. Informed consent was obtained. The study was approved by the Ethical Review Board of Beijing Anzhen Hospital and abides by the Declaration of Helsinki. This study is registered at ClinicalTrials.gov (ID: NCT03010514).

### Aortic ultrasonography monitoring

Images of the murine ascending aortas and aortic arches were captured using a micro-ultrasound system (VisualSonics Vevo 2100). The maximal aortic internal diameters were determined using Vevo 2100 software.

### Blood pressure measurement

SBP and DBP were measured using a noninvasive, tail-cuff analyzer (BP-2000 Blood Pressure Analysis System, Visitech Systems).

### Tissue preparation, histology, and immunostaining

Thoracic aorta samples (including the ascending and descending aortas) from BAPN-treated and Ang II-infused mice were fixed with 4% paraformaldehyde followed by processing in paraffinized or frozen sections. 7-μm-thick cross-sections were analyzed by H&E or EVG staining. For immunohistochemical staining, paraffin-embedded sections were incubated with 3% hydrogen peroxide, blocked with 3% blocking serum, incubated with the primary antibodies at 4 °C for 16 to 18 h, and secondary antibody at 25 °C for 45 min. The sections were then stained with a DAB kit, and the nuclei were counterstained with hematoxylin. Images were captured (Eclipse 90i digital microscopy system, Nikon) and analyzed (NIS-Elements Br 3.0). For immunofluorescence staining, the frozen sections were permeabilized with 0.3% Triton X-100, blocked with 10% goat serum, followed by incubation with the primary antibodies at 4 °C for 16 to 18 h and the Alexa Fluor 488 or Alexa Fluor 555 secondary antibody (Thermo Fisher Scientific) at 25 °C for 1 h. After 4′,6-diamidino-2-phenylindole mounting, images were captured (Olympus fluorescence microscope).

### Cell culture and transfection

HASMCs were obtained from ScienCell (#6110) and cultured in smooth muscle cell medium (SMCM basal medium ScienCell, #1001), supplemented with 20% fetal bovine serum, 1% smooth muscle cell growth supplement, and 1% penicillin-streptomycin. HEK293T cells were cultured in Dulbecco’s modified Eagle’s medium (Gibco) supplemented with 5% fetal bovine serum. Delivery of plasmids into HASMCs was performed *via* electroporation-based transfection (P1 Primary Cell 4D-Nucleofector X Kit, LONZA).

### Reverse transcription quantitative real-time PCR

Total RNA was extracted from thoracic aorta samples or HASMCs using TRIzol reagent (Invitrogen). Purified RNA (1–2 μg) was reverse transcribed into cDNA using Moloney murine leukemia virus reverse transcriptase (Promega). SYBR Green 2× PCR mix (Takara) was used for RT-qPCR, and the amplification reactions were performed using a Bio-Rad iCycler iQ5 (Bio-Rad). The relative expression levels of the target mRNAs were normalized to those of GAPDH. The primer information are listed in Table S1.

### Western blotting analysis

Proteins were extracted from aortic tissues by grinding them in liquid nitrogen, followed by the addition of tissue protein extraction reagent and a protease inhibitor cocktail (Thermo Fisher Scientific). Cell lysate supernatants were concentrated using a BCA protein assay kit (Thermo Fisher Scientific). Approximately 50 μg of protein was separated using SDS-PAGE and detected on polyvinylidene difluoride membranes after incubation with the primary antibody and the infrared dye 800CW-conjugated secondary antibody as described previously (31).

### Co-immunoprecipitation

To identify the binding proteins of KLF15, HEK293T cells were transfected with the Flag-KLF15 plasmid or Myc-KLF15 plasmid (control) for 30 h. Cells were lysed in a RIPA buffer and a protease inhibitor cocktail. The immunoprecipitation was performed using an ANTI-FLAG-M2 affinity gel (Sigma). Immunoprecipitated proteins were analyzed by SDS-PAGE and silver staining (Pierce Silver Stain Kit, Thermo). Stained proteins were excised for subsequent mass spectrometry analysis.

To confirm the interaction between endogenous KLF15 and MRTFB in HASMCs, the lysate was incubated with anti-MRTFB antibody or control IgG, followed by incubation with Protein A/G PLUS-beads (Santa Cruz). After three washes, the beads were analyzed by immunoblotting with anti-KLF15 and anti-MRTFB antibodies.

### Luciferase reporter assay

To analyze the effect of KLF15 knockdown on MRTFB-induced contractile gene expression in VSMCs, HASMCs were transfected with KLF15-targeting siRNAs or control siRNAs (si-NC). At 36 h post siRNA transfection, the cells were subjected to electroporation-based transfection of the MRTFB plasmid, α-SMA-Luc or SM22α-Luc reporter plasmid, and the pRL-TK control plasmid. Cell lysates were harvested to determine luciferase activity.

### Statistical analysis

All data are shown as the mean ± standard deviation. GraphPad Prism 7.0 was used to determine the statistical significance. The Shapiro-Wilk test was applied to check normality, with *p* >0.05 accepted as a normal distribution. Bartlett’s test was used to check for similar variances among normally distributed data, with *p* <0.05 considered unequal variances. The unpaired Student *t* test was used for comparison between two groups. For comparisons between more than two groups, one-way or two-way analysis of variance with Tukey’s post-hoc test was used. Fisher’s exact test was used to compare incidence of TAD. *p* <0.05 was considered statistically significant.

## Data availability

All data generated in this study are included in this published article.

## Supporting information

This article contains supporting information.

## Conflict of interest

The authors declare that they have no conflicts of interest with the contents of this article.
