# Supplementary material for: KLF15 maintains contractile phenotype of vascular smooth muscle cells and prevents thoracic aortic dissection by interacting with MRTFB
Source: J Biol Chem. 2024 Apr 4;300(5):107260. doi: 10.1016/j.jbc.2024.107260 (PMC11061230; doi:10.1016/j.jbc.2024.107260)
Supplement: Supporting Tables S1 and S2 [file mmc1.pdf]

Table S1 Oligonucleotides for RT-qPCR

Gene	GI	Forward Sequence (5'- 3')	Reverse Sequence (5'- 3')	Product Size (bp)	Tm (°C)
Klf15	1245897772	GTCAACATCCAGGGGCAGACCTT	TTGGCGGCAATGGGCACAG	110	62
Myh11	2211362176	GTGGAGAAGCTATGCTCAGAG	GTCACATTGTCATTTAGCGGGT	164	57
α-Sma	440309867	GAGGGATCCTGACGCTGAAGT	GCAGTGTCGGATGCTCTTCAG	129	59
Sm22α	291045204	CAGATGGAACAGGTGGCTC	CAGTTGGGATCTCCACGGT	185	57
Cnn1	226423896	GATGGCCTCAAAGACGGGAT	CGTTGGCCTCAAAGATGTCGT	175	58
Il-6	930945753	GGGAAATCGTGGAAATGAGA	TTCTGCAAGTGCATCATCGT	77	56
Ccl2	141803162	CTGTTCACAGTTGCCGGCT	CTACAGCTTCTTTGGGACACCT	163	59
Mmp2	985801704	CCAGCAAGTAGATGCTGCCT	GGCATCCAGGTTATCAGGGAT	164	58
Mmp9	2020930347	CCTGGAACTCACACGACATCT	CAGGAGGTCGTAGGTCACGT	155	59
Gapdh	281199965	AATGCATCCTGCACCACC	ATGCCAGTGAGCTTCCCG	248	58
KLF15	1519313919	TGTACACCAAAAGCAGCCAC	TCAGAGCGCGAGAACCTC	106	60
MYH11	1889454420	GATCGTCGACATGTACAAGGG	GGCCAGGTACTGAATGACCT	178	60
α-SMA	2239796742	CAGCTACGTGGGTGACGAAG	CAGGGTGGGATGCTCTTCAG	160	62

SM22α	1234543347	GATGGAGCAGGTGGCTCAGT	GATCTCCACGGTAGTGCCCAT	176	60
IL-6	1531243779	AGTGAGGAACAAGCCAGAGC	GTCAGGGGTGGTTATTGCAT	99	58
CCL2	1519313681	GCTCATAGCAGCCACCTTCAT	GACACTTGCTGCTGGTGATTC	146	58
MMP2	700274110	GATGTCCAGCGAGTGGATG	GCATCCAGGTTATCGGGGAT	167	57
MMP9	1519311730	GTTCGACGTGAAGGCGCAG	GTTCAACTCACTCCGGGAACT	166	60
GAPDH	53734501	AATGCCTCCTGCACCACC	ATGCCAGTGAGCTTCCCG	248	58

Table S2 Antibodies

Name	Application	Source
Anti-KLF15	IHC: 5 μg/ml<br>IF: 1:50<br>WB: 1: 500	NOVUS (NBP2-24635)<br>Invitrogen (MA5-15491)<br>Invitrogen (MA5-15491)
Anti-Myh11	WB: 1:500<br>IHC: 1:300	Servicebio (GB151220)<br>Servicebio (GB151220)
Anti-α-SMA	WB: 1:1000<br><br>IHC: 1:300<br>IF: 1:100	Servicebio (GB111364);<br>Abcam (ab119952)<br>Servicebio (GB111364)<br>Servicebio (GB111364)
Anti-SM22α	WB: 1:500	Servicebio (GB11366)
Anti-CD45	IHC: 1:300	Servicebio (GB113886)
Anti-CD68	IHC: 1:100	Servicebio (GB113109)
Anti-MMP2	IHC: 1:500	Servicebio (GB11130)
Anti-MMP9	IHC: 1:300	Servicebio (GB12132)

Anti-MRTFB	WB: 1:500<br>IF: 1:100<br>IP: 1μg	NOVUS (NBP1-46209)<br>NOVUS (NBP1-46209)<br>NOVUS (NBP1-46209)
Anti-Myc tag	WB: 1:1000	Invitrogen (PA1-981)
Anti-GAPDH	WB: 1:1000	Abcam (ab9485)
